# Recent advances in understanding dengue

**DOI:** 10.12688/f1000research.19197.1

**Published:** 2019-07-31

**Authors:** Scott Halstead

**Affiliations:** 1 Emeritus Professor, Department of Preventive Medicine and Biostatistics, Uniformed Services University of the Health Sciences, Bethesda, MD, 20814, USA

**Keywords:** dengue, flavivirus, pathogenesis, immunity, dengue fever, severe dengue, vaccines, epidemiology

## Abstract

This is a selective review of recent publications on dengue clinical features, epidemiology, pathogenesis, and vaccine development placed in a context of observations made over the past half century. Four dengue viruses (DENVs) are transmitted by urban cycle mosquitoes causing diseases whose nature and severity are influenced by interacting factors such as virus, age, immune status of the host, and human genetic variability. A phenomenon that controls the kinetics of DENV infection, antibody-dependent enhancement, best explains the correlation of the vascular permeability syndrome with second heterotypic DENV infections and infection in the presence of passively acquired antibodies. Based on growing evidence *in vivo* and *in vitro*, the tissue-damaging DENV non-structural protein 1 (NS1) is responsible for most of the pathophysiological features of severe dengue. This review considers the contribution of hemophagocytic histiocytosis syndrome to cases of severe dengue, the role of movement of humans in dengue epidemiology, and modeling and planning control programs and describes a country-wide survey for dengue infections in Bangladesh and efforts to learn what controls the clinical outcome of dengue infections. Progress and problems with three tetravalent live-attenuated vaccines are reviewed. Several research mysteries remain: why is the risk of severe disease during second heterotypic DENV infection so low, why is the onset of vascular permeability correlated with defervescence, and what are the crucial components of protective immunity?

## Introduction

Dengue viruses (DENVs) are relatively new pathogens. Transmission of virus from human to human by the bite of the mosquito *Aedes aegypti* began around three centuries ago ^1, 2^. Establishing an urban cycle required three separate emergence events: (a) evolution of a West African tree hole-breeding ancestor into *Aedes aegypti*, an anthropophilic domestic-breeding sub-species, and transportation of this mosquito (b) to tropical America via the slave trade and (c) in reverse direction to Europe and Asia where each of the four DENVs were introduced into the urban transmission cycle ^3, 4^. These emergence events in all likelihood were preceded by the circulation of a parental virus in sub-human primates throughout greater Southeast Asia during the Quaternary ice age ^5^. Oceans rose to isolate populations of dengue-infected sub-human primates on mainland Asia and on the islands of Indonesia and the Philippines ^6^. Viral evolution did the rest. Now these viruses cause a global pandemic, a consequence of jet airplane distribution of viremic humans throughout a tropical world comprehensively infested by *Aedes aegypti* ^7, 8^.

The world of dengue is massive and its features are constantly changing, as evidenced by the expanding scientific literature. Studies on dengue as an arthropod-borne viral disease contribute to three areas of research: (a) one on mosquito vectors, their bionomics, virus–host interactions, epidemiology, and control; (b) a literature on the virus itself; and (c) another describing DENV–human interactions. This review is directed to the last of these, focusing on clinical features, epidemiology, pathogenesis, and vaccine development.

The four DENVs are genetically related and biologically similar. Dengue is not the only human viral pathogen to circulate in biologically or genetically related groups. Many enteroviruses and respiratory viruses exist in groups of closely related pathogens. Although some degree of cross-protection may accompany sequential infections with members of these groups, most such outcomes are not well studied or understood. Dengue differs for researchers, a clear-eyed view of the observed complexity of dengue 1, 2, 3, and 4 (DENV) interactions with humans is crucial. For details of dengue disease features and pathogenesis, see my previous F1000 report ^9^. Infection with a single virus is followed by up to three outcomes: (1) durable protection against infection with a strain of the same DENV, (2) brief protection against infection or disease with a different dengue serotype, and (3) a breakthrough infection with a different dengue serotype that may result in severe disease ^10– 12^. This last outcome has a unique variant: dengue infection in the presence of passively acquired multitypic dengue antibodies may produce severe disease ^13^. Severe dengue in infants born to dengue-immune mothers is an important problem where dengue is highly endemic, causing 5% of dengue hospitalizations of children ^14– 17^.

## Clinical responses

Dengue clinical responses are subject to constraints imposed by infecting virus, epidemiology, human immune status, and human genetic makeup. These are reviewed briefly. Dengue disease severity differs by infection parity. Disease may accompany a first or second dengue infection and is infrequent during a third and absent during a fourth ^18– 20^.

### First infections

Intuitively, infection should result in a dengue clinical outcome. This is far from being the case. Age and viral type have an impact. In young children, primary dengue 1–4 infections are frequently inapparent ^21– 24^. As children age, disease response, particularly after puberty, becomes more adult-like. The infecting dengue type interacting with age also controls disease severity. In adults, primary dengue 1 and 3 infections result in high rates of classic dengue fever but dengue 2 and 4 infections cause milder disease and are often inapparent ^12, 25^. Primary dengue 2 and 4 infections in children of all ages frequently are inapparent ^24, 26^. Primary dengue 1 infections in children are modestly severe to the point of requiring hospitalization ^26^.

### Second infections

Second heterotypic dengue disease has been observed in 12 sequences ^19^. In children, overt disease is recognized clinically in perhaps 20 to 30% of second heterotypic dengue infections and severe disease in about 2% ^12, 27, 28^. A high incidence of severe disease has been reported for infection sequences with DENV1–2, 3–2, 4–2, 1–3, and 2–1 ^27, 29– 32^. The severity of second heterotypic dengue infections is controlled by age at the time of infection and the interval between first and second infections. Owing to their intrinsic risk of severe vascular permeability, young children are at higher risk of fatal outcome with heterotypic DENV infections ^33^. Exposure to DENV at intervals of less than 2 years may inhibit infection and suppress disease severity ^19, 34^. Short-term protection and ultimately enhanced second heterotypic DENV infection outcome may be controlled by natural declines or changes in heterotypic antibodies (or both) following a first DENV infection ^35, 36^. An increase in disease severity was observed when the interval between first and second infections was 20 years as opposed to 4 years ^30^. As an explanation, it was observed that severe disease at a long interval was correlated with an observed steady decrease in heterotypic antibody titers over a long period ^37, 38^. During secondary dengue infections, adults are at greater risk than children of bleeding whereas children are at greater risk than adults of vascular permeability ^39^. Human genetic inhomogeneity, particularly across ethnic groups, affects disease expression in a variety of ways ^40– 43^. The most notable genetic effect is the suppression of severe disease accompanying a second heterotypic dengue infection that has been observed in black patients in sub-Saharan Africa and is linked to a gene that regulates cellular dengue infection via lipid metabolism ^44^. A final pathophysiological constraint during secondary dengue infection is the fact that the onset of vascular permeability is correlated with defervescence. Mechanisms controlling these constraints are not well understood.

Despite a consensus on the importance of early recognition of the remediable dengue vascular permeability syndrome (DVPS) (fever, thrombocytopenia, abnormal hemostasis, elevated liver enzyme levels, hypoalbuminemia, complement activation, and vascular permeability), there is not wide agreement about how to define dengue disease ^45^. “Severe dengue” may include life-threatening conditions such as severe bleeding or impaired central nervous system, heart, or kidney functions in the absence of vascular permeability. This wide range of poorly defined and diverse pathophysiological endpoints may impede the early recognition and rapid and accurate assessment of fluid losses to be followed by accurate management of fluid resuscitation. Critically, research to identify pathophysiological mechanisms of the vascular permeability syndrome requires relevant and standardized case definitions ^46^.

A literature describing unusual clinical features or outcomes of dengue infections continues to grow at a record pace fueled by the hundreds of thousands of dengue cases that present yearly to sophisticated health-care systems. It is often difficult to place these cases into a pathogenic context because of missing viral, immunological, or epidemiological details (see above). Needed are virus type, age and sex of patients, and infection parity (for example, IgM/IgG ratio) and, if there is a second heterotypic infection, evidence concerning the interval between first and second infections and the identity of first infecting DENV type.

A subset of severe dengue cases, hemophagocytic lymphohistiocytosis (HLH), is increasingly recognized. HLH, a rare and potentially fatal disorder associated with acute infections, is characterized by fever, pancytopenia, hepatosplenomegaly, and increased serum ferritin. In Puerto Rico, 22 dengue HLH cases, including one death, were detected from 2008 to 2013. Cases were often infants, who frequently had influenza co-infections and were satisfactorily treated with steroids ^47^. There have been a number of individual case reports of HLH, predominantly from Asia ^48^. Many of these occurred after prolonged fever ^49^. An early diagnosis of HLH could deflect from a careful search for vascular permeability and promote the premature use of steroids that have been widely but ineffectively used to treat vascular permeability shock syndrome ^50^.

Thrombocytopenia accompanies many dengue infections. The widely used term “dengue hemorrhagic fever” has sensitized doctors and patients alike to the possibility that severe life-threatening bleeding may occur without warning. For this reason, low platelet counts are widely considered to be an indication for the prophylactic administration of platelets. Clinical experts have uniformly advocated against this expensive and potentially dangerous practice ^51– 54^. Investigators in Singapore and Malaysia conducted an open-label, randomized, superiority trial of platelet transfusion and enrolled 372 persons (21 years old or older) with acute laboratory-confirmed dengue with thrombocytopenia (not more than 20,000 platelets per microliter) but no severe or persistent mild bleeding. After enrollment, the subsequent frequency of clinical bleeding was slightly higher (26%) in the transfusion group, 13 of whom had adverse events, than in controls. No deaths were reported ^55^.

Progress is being made in identifying and interpreting physiological signals in patients who may progress to shock syndrome. A low echocardiographic stroke volume index and reduced left ventricular function plus high admission venous lactate levels identified dengue patients at high risk of recurrent shock and respiratory distress ^56^. In 2000, age of 5 to 15 years, a history of vomiting, higher temperature, a palpable liver, and a lower platelet count were risk factors for dengue shock while, during illness, absolute values and rate of daily decline of platelet counts successfully predicted shock ^57^.

## Epidemiology

During yellow fever epidemics, it was well understood that multiple cases occurred among members of households but also in persons who visited infested homes. Though less obviously, this same epidemiological feature applies to dengue. Careful prospective studies in the city of Iquitos on the Amazon River in Peru successfully tracked household members throughout the city during DENV transmission seasons ^58^. For individuals, the risk of DENV infection is controlled by the presence or absence of *Aedes aegypti* in places visited during the daytime. Risk is not a matter of vector abundance but of vector presence. Individuals increased their estimated transmission rate from 1.3 if they stayed at home to 3.75 when they visited other locations during daytime hours (for example, markets or homes of friends or relatives) ^59^. DENV is not spread significantly by sick individuals. Those who developed dengue fever spent more time at home, visited fewer locations, and in some cases visited locations closer to home and spent less time at certain types of locations than did individuals who were well ^60^.

In highly endemic southern Vietnam and Yogyakarta, a city in Indonesia, mobility data collected from children and young adults via prospective travel diaries found that all ages spent about half of their daytime hours (5 a.m. to 9 p.m.) at home while children under the age of 14 years spent a greater proportion of their time within 500 m of home than did older respondents. Mobility of specific age groups within populations must be taken into consideration in planning dengue preventive interventions ^61, 62^.

There is now better understanding of how DENVs successfully maintain transmission when only 20 to 30% of infected persons develop a disease. Studies indicate that silent infections may contribute as much as 84% of total DENV transmission. In persons who do develop dengue disease, most infections of mosquitoes occur prior to the onset of symptoms and only 1% of mosquito infections occur after symptoms have begun ^63^. The substantial role that inapparent infections play during dengue epidemics may contribute to more rapid transmission and widespread geographic spread of virus, reducing the usefulness of case data to predict where an outbreak will occur and what its final size will be ^64, 65^.

The introduction of a new flavivirus, Zika, into the Western Hemisphere in 2015 was the cause of considerable consternation, not simply because of its unexpected linkage to Guillain–Barré syndrome; Zika infection of pregnant women also caused a congenital Zika syndrome in infants. There were additional fears that Zika infections might enhance DENV infections or vice versa. An entirely unsuspected outcome has been that Zika behaves like a highly effective dengue vaccine. The Zika epidemic of 2015 to 2016 was followed by a reduction in clinical dengue cases throughout Latin America from 2,413,693 in 2015 to about 500,000 in both 2017 and 2018, a 77% reduction in dengue cases (Pan American Health Organization data). Similar outcomes were reported from surveillance studies in Salvador, Brazil ^66, 67^. It is suspected that Zika infection may have served to protect an epidemiologically and clinically important group, monotypic dengue-immunes, responsible for secondary DENV clinical disease ^68^.

A mystery in the global dengue pandemic is why, in the mid-20th century, severe dengue was highly endemic in Southeast but not South Asia ^69^. A remarkable nationwide survey of vector mosquitoes, dengue antibodies, and clinical disease, conducted in Bangladesh, may provide an answer. Out of 97,162 total communities, 70 were randomly selected for visits in 2014 and 2016, and one sixth of households, including 5,866 individuals, were interviewed ^70^. Many areas of rural Bangladesh were found to be devoid of dengue disease with low prevalence of dengue antibodies and low or no populations of *Aedes aegypti* and *Aedes albopictus*. Residents of Dhaka and Chittagong, where a number of recent outbreaks of dengue and chikungunya have occurred, had a high prevalence of dengue antibodies, abundant vector mosquitoes, and disease. A cluster of smaller cities and villages in the Southwestern corner of the country had modest dengue endemicity. The epidemiology of dengue in India may have been similar to that in Bangladesh today but suffered a steady invasion of *Aedes aegypti* throughout the country and into rural areas. This epidemiological evolution may explain the recent emergence of epidemic severe dengue throughout India.

The reason why yellow fever continues to be a major health problem in the American tropics is a fateful event that occurred centuries ago: the stable introduction of yellow fever virus into a complex zoonotic cycle ^71^. All four DENVs circulate as zoonoses in Asia and zoonotic dengue 2 was possibly transplanted from Asia to Africa. Genetic studies suggest that, during the slave trade, the African DENV2 was brought to the American tropics, where it entered the urban cycle ^5^. But has a dengue zoonotic cycle been established? Only modest efforts have been made to answer this question. A search for a sylvatic DENV cycle was initiated in a forested area of the eastern Amazon near Iquitos, Peru, a city where all four DENVs are endemic. Twenty seronegative Aotus monkeys were kept in jungle locations for a total of 10 years and were bled for virus and for antibody conversions with no evidence of DENV ^72^.

## Pathogenesis

Soon after the DVPS was identified, clinical and epidemiological data strongly associated it with second heterotypic DENV infections and also with primary DENV infections of infants born to dengue-immune mothers ^11, 73^. Pathology studies have consistently demonstrated human DENV infection target cells to be of myeloid lineage ^74^. When DENV infections occur *in vitro* or *in vivo* in the presence of sub-neutralizing dengue antibodies, enhanced infections/disease may result ^35, 75^. Indeed, the peak of early illness viremias or antigenemia successfully predicted disease severity ^26, 76, 77^. This phenomenon, antibody-dependent enhancement (ADE) of DENV infection of Fc receptor–bearing cells, differs from infection of these same cells in the absence of antibodies by two mechanisms: an increase in the number of cells infected (extrinsic ADE) or an increase in the intracellular production of DENV (intrinsic ADE) ^78^.

DENV ADE has been observed in animal models. Second heterotypic DENV2 infections produced enhanced viremia but not vascular permeability in rhesus monkeys ^79^. Efforts to reliably achieve vascular permeability disease during second heterotypic DENV infections in animals, including mice, have not been successful ^80^. When DENV antibodies were passively transferred to rhesus monkeys prior to infection with DENV2, enhanced viremias were observed but without vascular permeability. Administration of monoclonal or polyclonal dengue antibodies to mice has regularly resulted in enhanced DENV infections accompanied by vascular permeability and other features of DVPS ^81^. Also, it has been possible to produce vascular permeability in DENV-infected infant mice born to dengue-immune mothers ^82^. But other hypotheses of severe dengue pathogenesis that attribute disease (1) to a weakened ability of secondary T cells to contain DENV infection because of the original antigenic sin phenomenon, (2) to the hyper-production of endothelium-damaging secondary infection T-cell cytokines and chemokines, (3) to the DENV non-structural protein 1 (NS1) heterophile antibodies raised during first DENV infections that damage platelets, endothelial cells, or blood clotting proteins during second infections, or (4) to the ability of dengue IgG immune complexes to stimulate mast cells to release vasoactive amines fail to satisfy the requirements of Occam’s razor ^83– 86^. None of these hypotheses explains the phenomenon of infant DVPS where B- and T-cell responses are primary and anti-DENV or anti-NS1 IgG antibody concentrations at the onset of illness are absent or very low.

ADE is a kinetic force of infection but by itself is not a direct cause of pathology. Recent work in several laboratories has uncovered a pathogen that mediates DVPS with enhanced infections that occur with actively or passively acquired dengue antibodies. It was long known that fatal DENV infections of mice could be prevented by anti-NS1 ^87^. NS1 is produced during all four DENV infections as well as those of other pathogenic flaviviruses ^88^. Instead of remaining cell-bound, dengue NS1 is released as a hexamer circulating in great quantities in acute-phase blood ^89^. A perceptive study, published in 2006, suggested that the high levels of circulating NS1 documented in patients with severe dengue might activate complement to mediate vascular permeability ^90^. Dengue NS1 has been shown to activate complement by the alternative pathway, target liver cells promoting intracellular DENV infection, complex with thrombin in acute-phase blood of severe dengue, activate platelets *in vitro* via Toll-like-receptor 4 (TLR4), and produce thrombocytopenia in TLR4 knockout and normal mice ^90– 93^. In 2015, an analogy between the cellular biology of bacterial lipopolysaccharides (LPSs) and that of DENV NS1 was discovered ^94^. Each interacts with TLR4 on the surface of monocytes, macrophages, and endothelial cells, inducing the release of a range of cytokines and chemokines. Some of these same cytokines and chemokines have been found in the blood of patients accompanying DVPS. *In vitro*, NS1 disrupted endothelial cell monolayer integrity. The authors concluded that DVPS was a viral protein toxicosis. NS1-mediated cytokine release could be inhibited by the TLR4 antagonist LPS– *Rhodobacter sphaeroides*, suggesting an avenue for therapeutic intervention.

Crucially, the same observation was confirmed in an *in vivo* model ^95^. DENV2 NS1 inoculated intravenously at physiologically relevant concentrations in sub-lethally DENV2-infected C57BL/6 mice resulted in lethal vascular permeability. Vaccination of mice with DENV2 NS1 protected them against endothelial leakage and death from lethal DENV2 challenge. Mice immunized with all four DENV NS1 proteins were completely protected against homologous DENV challenges, whereas immunization with DENV1 NS1 partially protected against heterologous DENV2 challenge. Furthermore, DENV NS1 was shown to directly alter the barrier function of pulmonary endothelial cell monolayers through disruption of the endothelial glycocalyx-like layer (EGL) by triggering the activation of endothelial sialidases, cathepsin L, and heparanase, enzymes responsible for degrading sialic acid and heparan sulfate proteoglycans ^96^. Separately, disruption of endothelial glycocalyx components had been shown to correlate with plasma leakage during severe DENV infection in humans ^97, 98^. More recently, the contribution of these DENV NS1-induced endothelial cell–intrinsic pathways to NS1-mediated vascular leakage was demonstrated to be independent of inflammatory cytokines but dependent on the integrity of endothelial glycocalyx components both *in vitro* and *in vivo* ^99^. A DENV NS1 vaccine is taking shape ^100^. In conclusion, NS1 toxicosis is demonstrably an efferent mechanism of vascular permeability in mice and is controlled by DENV production in cells and, in turn, by ADE ^88^.

As described, DVPS is a rare outcome of a second heterotypic DENV infection. Identifying those at risk and understanding why they develop overt versus silent infections continue to be focuses of research. In a long-standing children’s cohort in Kamphaeng Phet, Thailand, peripheral blood mononuclear cells (PBMCs) were collected from children prior to their experiencing second heterotypic DENV infections, whether overt or silent. Cultured pre-infection PBMCs were stimulated with DENV1–4 antigens. Of 30 cytokines or chemokines studied, six had elevated responses in children who experienced silent second DENV infections and three had elevated responses in children who developed symptoms during a subsequent DENV infection. Significant differences were found in cytokine production based on both the type of DENV used for stimulation and the occurrence of clinical illness. Secretion of interleukin 15 (IL-15) and monocyte chemotactic protein 1 (MCP-1) was significantly higher by PBMCs of subjects who later developed symptomatic DENV infection. These studies are beginning to show how genetic and metabolic phenomena differing between individuals may control infectious processes and host responses ^101^.

## Vaccines

### Dengvaxia

The most advanced dengue vaccine is a chimera of structural genes of the four DENVs with the yellow fever vaccine non-structural genome. This vaccine was tested for vaccine efficacy and safety in placebo-controlled clinical trials enrolling 35,000 children (2 to 16 years old) in 10 dengue-endemic countries ^102^. Efficacy results were mixed. Through year 3 after the first dose, vaccine protection against hospitalization of children at least 9 years old was 65.5% but among children 8 years old or younger was 44.6% ^102^. In the 2- to 5-year-old age group, vaccinated children were hospitalized five times more frequently than those in the placebo group. Among the 11% of children whose serostatus (when vaccinated) was known, protection in seronegative children 8 years old or younger was 14.4% but in those 9 years old or older was 52.5%. These data led the manufacturer and expert committees to recommend that vaccine be restricted to children 9 years old or older ^102, 103^. A new serological test made it possible to distinguish those who were seronegative from those who were dengue-immune at the time of vaccination ^104^. When this test was applied to sera from a 10% randomized cohort of phase 3 children, dengue seronegativity rather than age controlled risk to severe hospitalized dengue disease (platelet count of less than 100,000 mm ^3 ^evidence of vascular permeability) over the period 5 to 6 years after first dose in children given vaccine ^105^. With this evidence of declining efficacy, the World Health Organization (WHO) Scientific Advisory Group of Experts, the Global Advisory Committee on Vaccine Safety, and the WHO Dengue Vaccine Working Group in 2016 recommended that vaccine be given to individuals with known past dengue infection or to populations with 80% DENV seroprevalence ^106^.

Why did Dengvaxia fail to protect seronegative children? When vaccinated children developed dengue disease despite circulating tetravalent DENV neutralizing antibodies, this provided solid evidence that conventionally measured human neutralizing antibodies were not protective ^102, 107– 110^. New observations suggest that it may be necessary to redefine the design of classic neutralization tests. When live DENV1 virus recovered from humans during the acute phase of a dengue infection was used to measure neutralization by antibodies, this virus was neutralized only by homotypic antibodies. By contrast, DENV1 grown in C6/36 or Vero tissue cultures was highly neutralized by homotypic and heterotypic dengue antibodies. Why? DENV1 grown in humans was found to be fully mature and 50- to 700-fold more infectious in cell culture than virus harvested after one passage in C6/36 or Vero cells. Human plasma and cell culture–derived DENV1 virions had identical genome sequences, indicating that differences in the neutralization of virus were attributable to the maturation state ^111^. Might DENV maturation status affect biological outcomes of DENV–antibody interactions, such as heterotypic protection or ADE?

Another explanation for Dengvaxia protection failure is that antibodies raised by vaccine may be poorly matched to the specific DENV genotypes in circulation. Two groups found genetic differences in the DENVs recovered during the first 25 months after first dose from placebos or vaccinated children ^112, 113^. The DENV4 in the Sanofi vaccine is genotype II. Genotype I (GI) and genotype II (GII) DENVs were both circulating in Asia during the vaccine trial. Both groups found vaccine efficacy to be higher against GII (83%) compared with GI (47%) DENV4s. In fact, Juraska and colleagues demonstrated that variations at three positions on the envelope protein of DENV4 are strongly correlated with vaccine efficacy ^112^. These three amino acid positions map to a region on E protein recognized by strongly neutralizing antibodies in people who have been infected with DENV4 ^114^. Indeed, another study recently demonstrated large differences in the ability of sera from naïve subjects who received the National Institutes of Health dengue vaccine (also based on a DENV4 GII envelope) to neutralize different genotypes of DENV4 ^115^. When Dengvaxia data were stratified by age, the effect was stronger in younger than older children ^112^. In children 2 to 8 years old, vaccine efficacy against GII viruses was 76.3% whereas efficacy against GI viruses was only 23.9%. In older children (9 to 14 years old), the vaccine was highly efficacious against both GII (89.8%) and GI (85.5%) viruses. The observation that older children who received the Sanofi vaccine were equally protected against DENV4 GI and GII viruses is best explained by the fact that most of these children had immunity to DENVs before vaccination. In this population, the vaccine stimulates strongly cross-neutralizing and cross-protective antibodies analogous to antibodies that develop after natural second DENV infections with a heterologous serotype ^116^. These antibodies, which target conserved epitopes between serotypes, are unlikely to be influenced by subtle differences between genotypes. In people with no pre-existing immunity to DENVs, current vaccination strategies rely on the ability to induce serotype-specific protection. However, Dengvaxia protection is not durable. Indeed, over the period of 5 to 6 years after first dose, children 2 to 8 years old, vaccinated when seronegative, were not protected but neither did they experience enhanced DENV4 disease ^105^. It is known that Dengvaxia raises DENV4 type-specific neutralizing antibodies in seronegative persons ^116^. Perhaps, early after vaccination, DENV4 GI antibodies circulate at levels that protect against vaccine-matched GII but not the mismatched GI strains.

Another issue is that T-cell immunity may be crucial to durable protection. Studies on dengue-immune humans in Sri Lanka found that multifunctional CD8 ^+^ T-cell responses were correlated with protection from DENV disease ^117^. Functional CD8 ^+^ T-cell responses were directed at a distinct pattern of non-structural protein antigens ^118^. There is growing evidence that human T-cell responses directed at epitopes on non-structural proteins contribute importantly to homotypic and heterotypic DENV protective immunity ^118, 119^. These observations were extended to human CD4 ^+^ T cells that are primed by DENV capsid, NS3 and NS5 antigens ^120, 121^. CD4 ^+^ and CD8 ^+^ T cells raised after a single dose of a tetravalent live-attenuated dengue vaccine were directed to the same repertoire of non-structural protein antigens as T cells from naturally infected humans ^122, 123^. A high frequency of CD107a ^+^ IFN-γ ^+^ CD8 ^+^ T cells raised in A 129 mice immunized with DENV2 PDK53 live-attenuated vaccine mediated efficient viral clearance and superior protection against wild-type DENV challenge ^124^. Dengvaxia does not present non-structural DENV proteins to the immune system. Might this absence contribute to the ineffective vaccine protection of seronegatives?

### TAK 003

In January 2018, the Takeda Pharmaceutical Company (Tokyo, Japan) announced the completion of phase 3 trials for TAK 003 ^125– 128^. Efficacy and safety data for this vaccine have not yet been published ( https://www.takeda.com/newsroom/newsreleases/2019/takedas-dengue-vaccine-candidate-meets-primary-endpoint-in-pivotal-phase-3-efficacy-trial/). The vaccine consists of a live-attenuated DENV2 strain and three chimeric viruses containing the prM and E protein genes of DENV1, 3, and 4 expressed on the backbone of the DENV2 genome ^129, 130^. Of these four viruses, one is a successful vaccine candidate, DENV2 16881 PDK 53. This virus achieved exceptionally high rates of seroconversions in seronegative human volunteers with minimal dengue signs or symptoms ^131^. Attenuating mutations for all four DENVs were identified, and infectious cDNA clones constructed ^132, 133^. The vaccine also includes structural DENV1, 3, and 4 proteins expressed on a DENV2 backbone. The developers hope for successful protection against all DENV infection/disease on the basis of the broad neutralizing antibody responses that follow two doses of TAK 003. Publication of clinical data from phase 3 clinical trials is awaited with interest.

### Live-attenuated tetravalent dengue vaccine

There is good dengue vaccine news. For nearly 20 years, the National Institute of Allergy and Infectious Diseases and the Johns Hopkins Bloomberg School of Public Health have designed and tested dengue vaccine candidates. Some were attenuated by removing nucleotides from the non-translated region of the dengue genome. A crucial component of this development program was testing monovalent vaccine candidates for immunogenicity and attenuation in seronegative human volunteers ^134^. A final product, the live-attenuated tetravalent dengue vaccine (LATV), consists of mutated DENV 1, 3 and 4 and a chimera of structural DENV 2 on a DENV 4 backbone ^135– 137^. Following a single dose of LATV, volunteers were solidly protected from viremia, dengue symptoms, or anamnestic antibody responses after challenge with non-parental wild strains of live DENV2 (Tonga 74) or 3 (Sleman 78) ^138^ (Dr. Anna Durbin, personal communication, April 4, 2019). That this protection is likely to be of long duration is evidenced by the solid immune response observed to a booster dose of live-attenuated vaccine given 12 months after initial dose ^139^. Protection results are complemented by evidence that a single dose of LATV raises monospecific neutralizing antibodies that are conformationally similar to antibodies raised after human infections with wild-type DENVs that correlate with protection ^140– 142^. Moreover, the T-cell responses to LATV closely resemble those raised after infections with wild-type DENVs ^122, 123^. Finally, LATV contains genes for three of the four DENV NS1 proteins. LATV is in the third year of phase 3 clinical testing in Brazil. The rate of accruing dengue vaccine efficacy data has been delayed because of an 80% reduction of DENV cases following the Zika virus epidemic of 2016–17 in Brazil ^143^. The outlook for LATV, based on phase 2 clinical trials in humans, is that a single dose of this vaccine will raise durable and solid protection against dengue infections in both seronegatives and seropositives.
